# Increased Serum LIGHT Levels Correlate with Disease Progression and Severity of Interstitial Pneumonia in Patients with Dermatomyositis: A Case Control Study

**DOI:** 10.1371/journal.pone.0140117

**Published:** 2015-10-08

**Authors:** Takuya Kotani, Tohru Takeuchi, Takaaki Ishida, Ryota Masutani, Kentaro Isoda, Kenichiro Hata, Shuzo Yoshida, Shigeki Makino, Toshiaki Hanafusa

**Affiliations:** 1 Department of Internal Medicine (I), Osaka Medical College, Takatsuki, Osaka, Japan; 2 Department of Central Laboratory, Osaka Medical College, Takatsuki, Osaka, Japan; Universitatsklinikum Freiburg, GERMANY

## Abstract

**Background:**

Activated CD8+ T cells play an important role in the pathogenesis of dermatomyositis (DM) with interstitial pneumonia (IP). Serum CD8+ T-cell activator, LIGHT, and Th1/Th2/Th17 cytokines were measured in DM-IP patients and compared with clinical parameters to investigate their usefulness.

**Methods:**

The correlations between the clinical findings and serum LIGHT and Th1/Th2/Th17 cytokine levels were investigated in 21 patients with DM-IP (14 with rapidly progressive IP [RPIP] and 7 with chronic IP [CIP], including 4 fatal cases of IP).

**Results:**

The median serum LIGHT level was 119 (16–335.4) pg/ml, which was higher than that in healthy control subjects and DM patients without IP. The median serum IL–6 level was 14.7 (2.4–154.5) pg/ml (n = 13). The other cytokines were detected in only a few patients. The median serum LIGHT level in DM-RPIP patients (156 [49.6–335.4] pg/ml) was significantly higher than that in DM-CIP patients (94.3 [16–164.2] pg/ml) (*P* = 0.02). The serum IL–6 level did not correlate with either progression or outcome of DM-IP. ROC curve analysis determined a serum LIGHT level of ≥120 pg/ml to be the cut-off value for the rapid progression of DM-IP. Serum LIGHT levels correlated significantly with %DLco (*R* = 0.55, *P* = 0.04) and total ground-glass opacity scores (*R* = 0.72, *P* = 0.0002). The serum LIGHT level significantly decreased to 100.5 (12.4–259.3) pg/ml 4 weeks after treatment initiation (*P* = 0.04).

**Conclusions:**

The serum LIGHT level may be a promising marker of disease progression and severity in patients with DM-IP.

## Introduction

Dermatomyositis (DM) is frequently complicated with interstitial pneumonia (IP), causing increased morbidity and mortality [1, 2]. CD8+ T and Th1 cells play important roles in the pathogenesis of IP [3, 4]. On the basis of their clinical course, DM patients with IP are classified into one of two groups: DM with rapidly progressive IP (RPIP) or DM with chronic IP (CIP). DM-CIP slowly progresses and the prognosis is favorable. In contrast, DM-RPIP progresses within several weeks to 3 months, and aggressive combination therapy with corticosteroids and immunosuppressive drugs such as calcineurin inhibitors and intravenous pulse cyclophosphamide (IVCY) needs to be performed [5–8]. Thus, it is important to identify a useful serum marker associated with the progression, severity, and prognosis of DM-IP patients.

LIGHT (the name of which is derived from “homologous to lymphotoxins, exhibits inducible expression, competes with herpes simplex virus glycoprotein D for herpes simplex virus entry mediator [HVEM], and expressed by T lymphocytes”) is a member of the TNF superfamily and activates CD4+ and CD8+ T cells, monocytes and macrophages, natural killer cells, immature dendritic cells, and platelets [9–13]. LIGHT mainly binds to the HVEM receptor on T cells and transmits co-stimulatory signals [14]. HVEM signals stimulated by LIGHT more strongly induce the activation of CD8+ T cells than CD4+ T cells [15]. Serum LIGHT has been reported as a potential biomarker of inflammatory diseases, such as rheumatid arthritis, ankylosing spondylarthritis, inflammatory bowel disease, and atopic dermatitis [16–19], but its association with the pathogenesis of DM-IP has not been clarified.

In this study, we compared serum LIGHT levels among patients with DM-IP or DM and healthy control subjects (HC) and investigated treatment-induced changes in the serum LIGHT levels of DM-IP patients. Th1/Th2/Th17 cytokines were also measured in these patients, and the correlation of these cytokines and serum LIGHT levels with other clinical parameters was compared to investigate their usefulness as a marker of disease progression and severity in DM-IP patients.

## Materials and Methods

### Study design

This retrospective study included patients with DM who were admitted to Osaka Medical College Hospital between April 2011 and March 2014. DM was diagnosed according to the criteria of Bohan and Peter [20, 21]. Clinically amyopathic DM (CADM) was diagnosed according to the criteria proposed by Sontheimer [22] and Gerami et al. [23]. Patients with an overlapping syndrome, such as systemic lupus erythematosus, systemic sclerosis, or malignancy, were excluded. IP was diagnosed by high-resolution computed tomography (HRCT) of the chest. RPIP was defined as IP with a respiratory condition, laboratory findings, arterial gas findings, chest HRCT scans, and pulmonary function test findings rapidly exacerbating within a period of days to 3 months after disease onset. Patients with CIP did not meet the definition of RPIP [24]. Patients’ clinical and laboratory findings were obtained from medical records at hospital admission.

### Ethics Statement

This study was approved by the ethical committee of Osaka Medical College (No. 1316) and complied with the guidelines of the Declaration of Helsinki. Written informed consent was obtained from each patient.

### Treatment

The administration of 0.75–1.0 mg/kg/day prednisolone was begun in all patients and was concomitantly administered with cyclosporine (CSA) or tacrolimus (TAC). The physicians decided which of CSA or TAC was used. CSA was initiated at 4 mg/kg/day once a day before breakfast, and the CSA level was adjusted at 2 hours after administration (C2), to 1500 ng/ml or above [8, 25]. TAC was initiated at 0.1 mg/kg/day twice a day before breakfast and dinner, and the trough level was adjusted to 10–20 ng/ml. The addition of IVCY was decided by physicians on the basis of the disease activity and patient condition.

### Measurement of laboratory parameters

The laboratory test items evaluated were creatine kinase (CK), lactic acid dehydrogenase (LD), C-reactive protein (CRP), Krebs von der Lungen–6 (KL–6), and ferritin. The upper limits of normal range for these tests were CK ≤ 200 IU/l, LD ≤ 250 IU/l, CRP ≤ 0.25 mg/dl, ferritin ≤ 465 ng/ml, and KL–6 ≤ 401 U/ml. Blood samples were collected from all patients at hospital admission, and the serum was stored at −20°C until measurement of the following cytokines and autoantibodies. Serum values of human LIGHT were assayed with an enzyme-linked immunosorbent assay (ELISA) kit (R&D Systems, USA). The detection limit of the serum LIGHT was 1.2 pg/ml. Serum values of human Th1/Th2/Th17 cytokines were assayed with a commercially available ELISA kit (Cytometric Bead Array Human Th1/Th2/Th17 Cytokine Kit; BD Biosciences, USA). Detection limits of the Th1/Th2/Th17 cytokines (IFN-γ, IL–2, TNF-α, IL–10, IL–4, IL–6, and IL–17) were 3.7, 2.6, 3.8, 4.5, 4.9, 2.4, and 18.9 pg/ml, respectively. Anti-melanoma differentiation-associated gene 5 (*MDA5*) antibody (Ab) was determined by ELISA using recombinant MDA5 antigen (OriGene, Rockville, MD, USA) as described previously [26]. Anti-aminoacyl-tRNA synthetase (ARS)-Ab was determined using a commercially available line blot test kit (Myositis Profile Euroline Blot test kit; Euroimmun, Lübeck, Germany).

### Arterial blood gas analysis and pulmonary function test

Arterial blood gas analysis including PaO_2_, PaCO_2_, and the alveolar-arterial oxygen difference (AaDO_2_) was performed on admission. Static and dynamic lung volumes were measured by spirometry (SYSTEM21; Minato Medical Science, Osaka, Japan). Vital capacity (VC) was determined by the N2 washout method. The diffusion capacity of the lung for carbon monoxide (DLco) was determined by the single-breath method. The results of pulmonary function testing are expressed as percentages of the predicted values.

### HRCT scoring

HRCT was performed using an Aquilon 64-detector row CT scanner (Toshiba Medical System Corporation, Tokyo, Japan) on admission. Slice thickness was 1.0–1.5 mm at 10-mm increments, which included the entire lung. In DM-IP patients, the disease activity of IP correlates with the range of ground-glass opacity (GGO) [27]. Therefore, the area of GGO was scored to assess HRCT findings, as previously described [28]. The area of GGO in each lobe (right upper, middle, and lower, and left upper and lower lobes) of the lung was scored on a scale of 0–5 at the 3 sites: mid aortic arch, tracheal carina, and 1 cm above the top of the right diaphragm. The scores were summed as the total CT score.

### Statistical analysis

Statistical analysis was performed using the Mann-Whitney U-test to compare median values and Fisher’s exact test to compare frequencies. Correlations were evaluated by using Spearman’s correlation coefficients. A value of *P* <0.05 was considered to indicate statistical significance. The data were analyzed using JMP software, version 11.0 (SAS Institute, Cary, NC, USA).

## Results

### Clinical characteristics of patients with DM-IP

Twenty-six patients with DM were enrolled in this study (Table 1), including 21 with IP (15 women; median age, 61 years, range 36–78 years) and 5 without IP (2 women; median age, 76 years, range 39–77 years). Five healthy control (HC) subjects were also enrolled (all women; median age, 55 years, range 50–68 years). In DM-IP patients, the frequencies of CADM, anti-MDA5-Ab positivity, and anti-ARS-Ab positivity were 67%, 38%, and 43%, respectively. The complications of RPIP and CIP were present in 14 (67%) and 7 (33%) patients, respectively. The median disease duration (from the onset of respiratory symptoms to the initiation of treatments) was 2.5 (interquartile range; 0.5–32) months. The values of CK, LD, CRP, KL–6, ferritin, and AaDO_2_ were 168 (17–6049) IU/ml, 330 (180–894) IU/ml, 0.95 (0.04–13.45) mg/dl, 1084 (423–3898) U/ml, 419.9 (28–23272.5) ng/ml, and 41.9 (5.9–113.6) mmHg, respectively. The value of the total CT score was 15 (10–23). The median dose of prednisolone was 50 (32.5–80) mg/day. CSA and TAC were administered to 13 and 7 patients, respectively. IVCY was additionally administered to 14 patients, and the median total dose was 1700 (300–4900) mg/body. Five patients died during a 48-week follow-up period, and the cause of death was IP-related respiratory failure in 4 and sepsis in 1. The median survival time of the patients who died of IP was 15 (7–37) weeks. The frequency of CADM in the DM non IP patients was 60%. The values of CK, LD, CRP, KL–6, and ferritin were 489 (62–1851) IU/ml, 251 (196–541) IU/ml, 0.2 (0.05–5.86) mg/dl, 187 (137–219) U/ml, and 287 (87.9–508.2) ng/ml, respectively. As of July 2015, of the patients who survived to 48 weeks after the institution of treatment, all maintained remission and no relapse of IP was observed.

**Table 1:** Clinical characteristics of the patients with DM-IP and DM without IP.

Characteristics	IP (n = 21)	Without IP (n = 5)
Age, years	61 (36–78)	76 (39–77)
Female, n (%)	15 (71)	2 (40)
CADM, n (%)	14 (67)	3 (60)
RPIP, n (%)	14 (67)	
Disease duration, months	2.5 (0.5–32)	
Positive anti-MDA5-Ab, n (%)	8 (38)	
Positive anti-ARS-Ab, n (%)	9 (43)	
CK, IU/l	168 (17–6049)	489 (62–1851)
LD, IU/l	330 (180–894)	251 (196–541)
CRP, mg/dl	0.95 (0.04–13.45)	0.2 (0.05–5.86)
KL–6, U/ml	1084 (423–3898)	187 (137–219)
Ferritin, ng/ml	419.9 (28–23272.5)	287 (87.9–508.2)
AaDO_2_, mmHg	41.9 (5.9–113.6)	
%VC	75.5 (48.1–93.1)	
%Dlco, ml/min/mmHg	32.7 (8.6–48.6)	
Total GGO score	15 (10–23)	
PSL (n = 21), mg/day	50 (32.5–80)	50 (45–50)^d^
CSA (n = 13), mg/day	250 (175–375)^a^	
TAC (n = 7), mg/day	3 (2–6)^b^	
Total IVCY (n = 14), mg	1700 (300–4900)^c^	
Fatal outcome due to IP, n (%)	4 (19)	

Values indicate the median (interquartile range). DM: dermatomyositis; IP: interstitial pneumonia; CADM: clinically amyopathic DM; RPIP: rapid progressive IP; Disease duration: disease duration from onset of respiratory symptoms of IP to initiation of treatments; MDA5: anti-melanoma differentiation-associated gene 5; Ab: antibody; ARS: aminoacyl-tRNA synthetase; CK: creatine kinase; LD: lactate dehydrogenase; CRP: C-reactive protein; AaDO_2_: alveolar-arterial oxygen difference; VC: vital capacity; Dlco: diffusion capacity of the lung for carbon monoxide; GGO: ground-glass opacity; PSL: prednisolone; CSA: cyclosporine; TAC: tacrolimus; IVCY: intravenous pulse cyclophosphamide.

^a^n = 13.

^b^n = 7.

^c^n = 14.

^d^n = 3.

### Levels of serum LIGHT and Th1/Th2/Th17 cytokines in DM-IP patients

**Fig 1:**
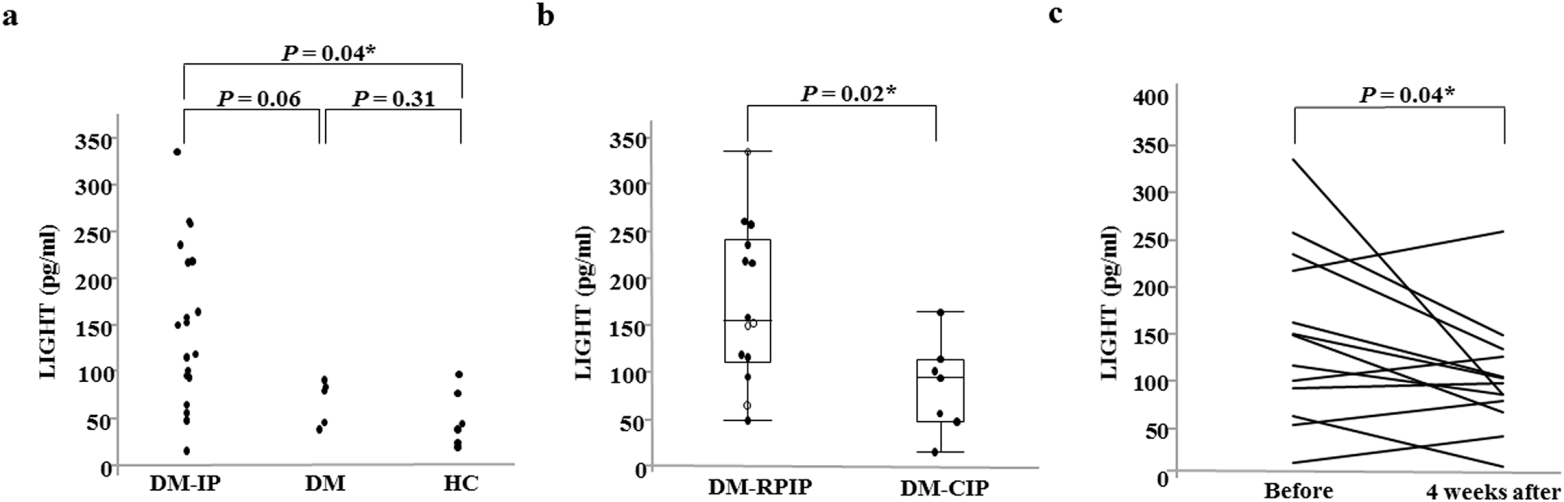
Serum LIGHT levels in patients with DM-IP (RPIP/CIP) or DM and the HC, and changes in serum LIGHT levels before and 4 weeks after treatment. The serum LIGHT level was higher in the DM-IP patients than in the DM patients and was significantly higher than that in the HC (a). The serum LIGHT level in the DM-RPIP patients was significantly higher than that in DM-CIP patients (b). The serum LIGHT levels at 4 weeks after treatment initiation were significantly decreased compared with those before treatment (c). DM: dermatomyositis; IP: interstitial pneumonia; HC: healthy control subjects; RPIP: rapidly progressive IP; CIP; chronic IP; Before: before treatment; 4 weeks after: 4 weeks after treatment. Open circle: dead due to IP; Closed circle: alive.

As shown in Fig 1a and Table 2, the median levels (range) of serum LIGHT were 119 (16–335.4) in DM-IP patients (n = 19), 80.4 (39.9–90.2) in DM patients (n = 5), and 41.7 (21–97.2) pg/ml in the age-matched HC (n = 5). The serum LIGHT level in the DM-IP patients was higher than that in the DM patients (*P* = 0.06) and HC (*P* = 0.04). No significant difference was noted in the serum LIGHT level between the DM patients and HC. The median level (range) of serum LIGHT was 218 (49.6–335.4) pg/ml in the DM-IP patients with high CK (> 200 IU/l, n = 9) and tended to be higher than that of 105.6 (16–261.3) pg/ml in the DM-IP patients with normal CK (≤ 200 IU/l, n = 12) (*P* = 0.06). The median serum IL–6 level was 14.7 (2.4–154.5) pg/ml, and it was undetectable in 8 patients. The other cytokines were detectable in only a few patients.

**Table 2:** Clinical characteristics, serum LIGHT levels, and Th1/Th2/Th17 cytokine levels in DM-IP patients.

No	Sex	Age years	Disease type	IP progression	anti-MDA5ab	anti-ARSab	LIGHT pg/ml	INF-γ pg/ml	IL–2 pg/ml	TNF pg/ml	IL–10 pg/ml	IL–4 pg/ml	IL–6 pg/ml	IL–17 pg/ml	Outcome
1	F	64	CADM	RPIP	Pos	Neg	150.1	28.7	ND	ND	5.3	ND	154.5	37.9	Dead
2	F	68	CADM	CIP	Neg	Pos	47.9	ND	ND	ND	ND	ND	ND	ND	Alive
3	M	52	CADM	RPIP	Pos	Neg	335.4	ND	ND	ND	ND	ND	22.7	ND	Dead
4	F	50	CADM	RPIP	Pos	Neg	159.3	ND	ND	ND	ND	ND	20.4	ND	Dead
5	M	77	DM	RPIP	Neg	Pos	218.7	ND	ND	ND	12.2	ND	74.5	ND	Dead*
6	M	57	CADM	RPIP	Neg	Pos	261.3	17.5	ND	ND	ND	ND	14.8	ND	Alive
7	F	73	DM	CIP	Neg	Neg	102.3	ND	ND	ND	ND	ND	2.5	ND	Alive
8	M	78	CADM	RPIP	Pos	Neg	65.3	ND	ND	ND	ND	ND	ND	ND	Dead
9	F	49	CADM	RPIP	Pos	Neg	152.6	ND	ND	ND	ND	ND	16.6	ND	Alive
10	F	68	DM	RPIP	Neg	Pos	119	ND	ND	ND	ND	ND	ND	ND	Alive
11	F	46	CADM	RPIP	Neg	Neg	116.7	ND	ND	ND	ND	ND	ND	ND	Alive
12	M	67	CADM	CIP	Neg	Neg	56.8	ND	ND	ND	ND	ND	ND	ND	Alive
13	F	61	CADM	CIP	Neg	Pos	94.3	ND	ND	ND	ND	ND	2.4	ND	Alive
14	M	67	DM	RPIP	Neg	Pos	258.3	ND	ND	ND	ND	ND	86.6	ND	Alive
15	F	77	DM	CIP	Neg	Neg	115.1	3.05	ND	ND	ND	ND	ND	ND	Alive
16	F	39	DM	RPIP	Neg	Pos	218	ND	ND	ND	ND	ND	12	ND	Alive
17	F	70	CADM	RPIP	Pos	Neg	96	ND	ND	ND	ND	ND	9.8	ND	Alive
18	F	51	DM	RPIP	Pos	Neg	49.6	ND	ND	ND	ND	ND	ND	ND	Alive
19	F	43	CADM	CIP	Pos	Pos	164.2	ND	ND	ND	ND	ND	7.9	ND	Alive
20	F	53	CADM	CIP	Neg	Neg	16	ND	ND	ND	ND	ND	ND	ND	Alive
21	F	36	CADM	RPIP	Neg	Pos	236	ND	ND	ND	ND	ND	4.5	ND	Alive

DM: dermatomyositis; IP: interstitial pneumonia; F: female; M: male; CADM: clinically amyopathic DM; RPIP: rapidly progressive IP; CIP: chronic IP; ND: not detected; Pos: positive; Neg: negative; Dead: dead due to IP.

*Dead due to septic shock.

### Comparison of serological biomarkers of DM-IP between the DM-RPIP and DM-CIP groups

No significant differences were noted in the anti-MDA5-Ab-positive rate, anti-ARS-Ab-positive rate, or CK, LD, KL–6, ferritin, and IL–6 levels between the DM-RPIP and DM-CIP groups (Table 3). The respective median levels (range) of serum CRP and LIGHT were significantly higher in the DM-RPIP patients, 1.95 (0.29–12.8) mg/dl and 156 (49.6–335.4) pg/ml, than in the DM-CIP patients, 0.14 (0.04–0.63) mg/dl and 94.3 (16–164.2) pg/ml, (*P* = 0.0004 and 0.02) (Fig 1b). To determine the cut-off point effective for determining the progression of DM-IP, receiver operating characteristic (ROC) curve analysis was carried out on the serum LIGHT levels. The value that maximized the area under the ROC curve was 116.7 pg/ml for serum LIGHT (sensitivity: 78.6%, specificity: 64.3%). From this result, a LIGHT level of ≥120 pg/ml was determined as the cut-off value for rapid progression of DM-IP.

**Table 3:** Serum biomarkers of DM-IP on admission in patients with RPIP and CIP.

Variables	RPIP (n = 14)	CIP (n = 7)	*P*
Positive anti-MDA5-Ab, n (%)	7 (50)	1 (14)	0.17
Positive anti-ARS-Ab, n (%)	6 (43)	3 (43)	1
CK, IU/l	287 (17–5330)	103 (77–6049)	0.91
LD, IU/l	416 (230–782)	253 (180–894)	0.23
CRP, mg/dl	1.95 (0.29–12.8)	0.14 (0.04–0.63)	0.0004*
KL–6, U/ml	1255.5 (425–3898)	1017 (423–2015)	0.22
Ferritin, ng/ml	1073.5 (28–23272.5)	97.6 (46.7–247.1)	0.28
LIGHT, pg/ml	156 (49.6–335.4)	94.3 (16–164.2)	0.02*
IL–6, pg/ml	18.5 (4.5–154.5)^a^	2.4 (0.6–7.9)^b^	0.08

Laboratory markers are presented as the median (interquartile range). DM: dermatomyositis; IP: interstitial pneumonia; RPIP: rapid progressive IP; CIP: chronic IP; MDA5: anti-melanoma differentiation-associated gene 5; Ab: antibody; ARS: aminoacyl-tRNA synthetase; CK: creatine kinase; LD: lactate dehydrogenase; CRP: C-reactive protein; IL–6: interleukin 6. The *P*-values were estimated using Fisher’s exact test or Mann-Whitney U-test.

*P <0.05.

^a^n = 10.

^b^n = 5.

### Comparison of serological biomarkers of DM-IP between the IP death and survivor groups

The anti-MDA5-Ab-positive rate was significantly higher in the IP death group (100%) than in the survivor group (25%) (*P* = 0.01), as shown in Table 4. No significant difference was noted in the anti-ARS-Ab-positive rate or the CK, LD, CRP, KL–6, ferritin, LIGHT, and IL–6 levels between these two groups.

**Table 4:** Serum biomarkers of DM-IP on admission in patients dead due to IP and those still alive.

Variables	Dead (n = 4)	Alive (n = 16)	*P*
Positive anti-MDA5ab, n (%)	4 (100)	4 (25)	0.01*
Positive anti-ARSab, n (%)	0 (0)	8 (50)	0.12
CK, IU/l	105 (43–287)	174.5 (17–6049)	0.37
LD, IU/l	349 (312–485)	310 (180–894)	0.76
CRP, mg/dl	1.30 (0.91–2.85)	0.7 (0.04–8.27)	0.42
KL–6, U/ml	1808 (501–3898)	1074 (423–3006)	0.29
Ferritin, ng/ml	1330.3 (477.7–1611)	141.9 (28–23272.5)	0.79
LIGHT, pg/ml	154.7 (65.3–335.4)	115.9 (16–261.3)	0.34
IL–6, pg/ml	22.7 (20.4–154.5)^a^	7.9 (0.6–86.6)^b^	0.13

Laboratory markers are presented as the median (interquartile range). DM, dermatomyositis; IP, interstitial pneumonia; Dead, dead due to IP; MDA5, anti-melanoma differentiation-associated gene 5; Ab, antibody; ARS, aminoacyl-tRNA synthetase; CK, creatine kinase; LD, lactate dehydrogenase; CRP, C-reactive protein; IL–6, interleukin 6. The *P*-values were estimated using Fisher’s exact test or Mann-Whitney U-test.

*P <0.05.

^a^n = 3.

^b^n = 11.

### Correlation between serum LIGHT levels and other disease activity indicators of DM-IP

**Fig 2:**
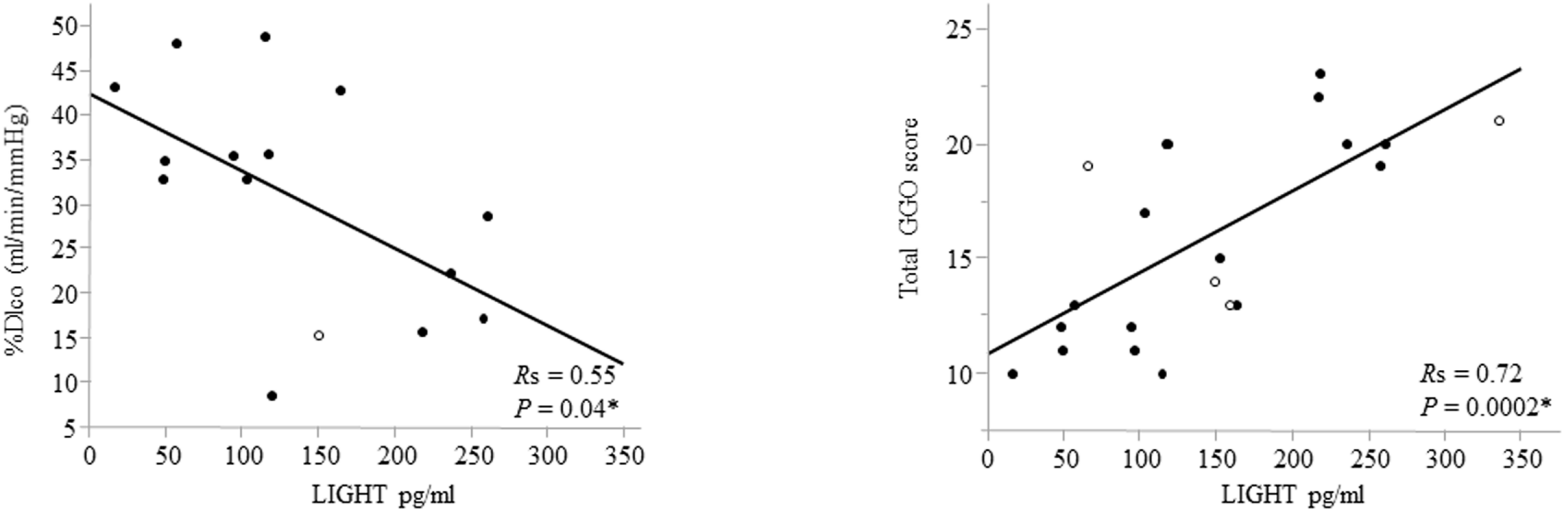
Correlation between serum LIGHT levels and %DLco and total GGO score of DM-IP. Serum LIGHT levels correlated significantly with %DLco (*R* = 0.55, *P* = 0.04) and total GGO scores (*R* = 0.72, *P* = 0.0002). DLco: diffusion capacity of the lung for carbon monoxide; GGO: ground-glass opacity; Open circle: dead due to IP; Closed circle: alive; *R*: correlation coefficient established using Spearman correlation coefficients. *P <0.05.

As shown in Table 5 and Fig 2, the LIGHT levels correlated significantly with LD (*R* = 0.45, *P* = 0.04), CRP (*R* = 0.58, *P* = 0.006), %DLco (*R* = 0.55, *P* = 0.03), and total GGO scores (*R* = 0.72, *P* = 0.0002). There was no correlation between the serum LIGHT levels and CK, KL–6, ferritin, AaDO_2_, and %VC values. The serum LIGHT levels were not associated with either the presence or absence of anti-MDA5-Ab (data not shown).

**Table 5:** Correlation between serum LIGHT levels and other disease activity indicators of DM-IP.

Variables	Correlation *R*	Analysis *P*
CK	0.19	0.41
LD	0.45	0.04*
CRP	0.58	0.006*
KL–6	0.27	0.24
Ferritin	0.25	0.27
AaDO_2_	0.37	0.10
%VC	0.41	0.12
%Dlco	0.55	0.03*
Total GGO score	0.72	0.0002*

DM: dermatomyositis; IP: interstitial pneumonia; CK: creatine kinase; LD: lactate dehydrogenase; CRP: C-reactive protein; AaDO_2_: alveolar-arterial oxygen difference; VC: vital capacity; Dlco: diffusion capacity of the lung for carbon monoxide; GGO: ground-glass opacity; *R*: correlation coefficient established using Spearman correlation coefficients.

*P < 0.05.

### Changes in serum LIGHT levels before and 4 weeks after treatment

The serum LIGHT levels were measured at 4 weeks after treatment initiation in 13 patients (Case Nos. 1, 3, 8–11, 13–15, 17, and 19–21). As shown in Fig 1c, the serum LIGHT levels (100.5 [12.4–259.3] pg/ml) at 4 weeks after treatment initiation were significantly decreased compared with those before treatment (150.1 [16–335.4] pg/ml) (*P* = 0.04).

## Discussion

The present study clarified that the serum LIGHT level was higher in the DM-IP than DM patients without IP and HC. In addition, the serum LIGHT level was significantly higher in the DM-RPIP than DM-CIP patients. Moreover, the serum LIGHT level correlated significantly with %DLco and the GGO score on chest CT before treatment, and decreased after treatment. This is the first report to suggest the use of serum LIGHT as a possible marker of disease progression and severity in patients with DM-IP.

Kurasawa et al. reported that activated Th1-type pulmonary T cells and CD25+CD8+ T cells play an important role in the development of corticosteroid-resistant IP in DM and polymyositis [29]. In DM-IP, lymphocyte infiltration in lung tissue, mainly that of CD8+ T and Th1 cells, is associated with the occurrence of an autoimmune response [30, 31]. Comparison between chest HRCT and histopathological findings of the lung revealed a close correlation of GGO with inflammatory cell infiltration in the lung interstitium [32]. Our study showed that the level of LIGHT, which activates CD8+ T cells, and the GGO score on chest HRCT were closely correlated, suggesting that the serum LIGHT level reflects the expansion of activated CD8+ T-cell infiltration in the lung interstitium.

LIGHT induces marked activation of CD8+ T cells and promotes Th1 cytokine production [15]. Gono et al. reported that the IL–10 and TNF-α levels before treatment were high (6.6 [1.6–13.8] and 16.8 [10.6–29.3] pg/ml, respectively) in 38 patients with polymyositis/DM-IP [33]. In our study, the levels of many Th1/Th2/Th17 cytokines excluding IL–6 were lower than the limits of detection. This inconsistency may have been due to differences in patient background and the measurement method of the cytokines.

A high KL–6 level, anti-MDA5 Ab positivity, high serum levels of ferritin, IL–6, IL–10, and IL–8, and a high level of AaDO_2_ have been reported as serological factors in DM-IP that indicate a poor prognosis [33–35, 36–39]. Sato et al. reported that anti-MDA5 Ab is a useful prognostic indicator of DM-IP [37]. In fact, the patients in all of the fatal cases in our study were anti-MDA5 Ab-positive. Nara et al. reported that the serum IL–6 level was 9 pg/ml or higher in all 6 patients with CADM-IP who died of IP, whereas it was lower than 9 ng/ml in 5 of 6 survivors [39]. It has also been reported that in patients with DM-IP, those with a serum ferritin level ≥ 1500 ng/ml, or those with a serum ferritin level ≥ 600 ng/ml plus AaDO_2_ ≥ 45 mmHg, have poor prognoses [8, 33]. In this study, the AaDO_2_ and serum ferritin, IL–6, and LIGHT levels were not associated with the outcome of DM-IP. This may have been due to the aggressive intervention with more potent immunosuppressive drugs from the early disease stage in patients with high levels of these markers.

**Fig 3:**
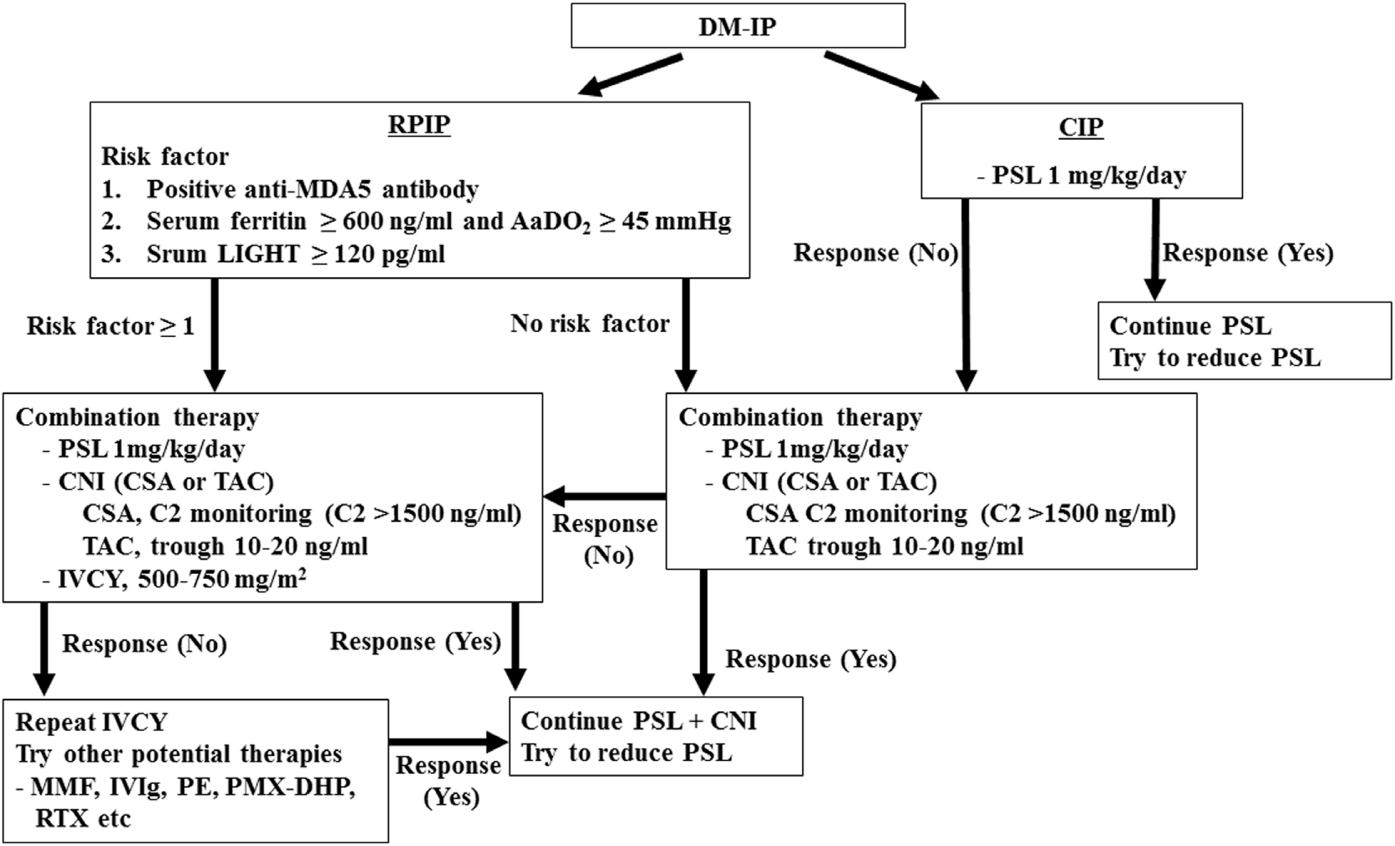
Therapy protocol for DM-IP patients. DM: dermatomyositis; IP: interstitial pneumonia; RPIP: rapidly progressive IP; CIP: chronic IP; MDA5: melanoma differentiation-associated gene 5; AaDO_2_: alveolar-arterial oxygen difference; PSL: prednisolone; CNI: calcineurin inhibitor; CSA: cyclosporine; C2: CSA level at 2 hours after administration; TAC: tacrolimus; IVCY: intravenous pulse cyclophosphamide; MMF: mycophenolate mofetil; IVIg: intravenous high-dose immunoglobulin; PE: plasma exchange; PMX-DHP: polymyxin B immobilized fiber column direct hemoperfusion; RTX; rituximab.

Based on the findings obtained in this study, our recommended therapeutic strategy for DM-IP is shown in Fig 3. Among patients with DM-RPIP, for those with any of the poor prognostic factors of anti-MDA5 Ab-positivity, serum ferritin ≥ 600 ng/ml plus AaDO_2_ ≥ 45 mmHg, or serum LIGHT ≥ 120 pg/ml, combined administration of prednisolone at 1 mg/kg/day and calcineurin inhibitor (CSA C2 level or TAC trough level adjusted to ≥ 1500 ng/ml or 10–20 ng/ml, respectively), and aggressive additional doses of IVCY are recommended as soon as possible after diagnosis.

## Conclusion

We propose that the serum LIGHT level may be a promising biomarker of disease progression and severity in patients with DM-IP. This was a retrospective study involving a small number of patients. The investigation of additional accumulated cases may clarify the usefulness of LIGHT as a prognostic factor of DM-IP, and thus further study is desired.
